# Congenital cystic adenomatoid malformation of lung in adults: 2 rare cases report and review of the literature

**DOI:** 10.1186/1746-1596-7-37

**Published:** 2012-04-03

**Authors:** Anning Feng, Hourong Cai, Qi Sun, Yifen Zhang, Lulu Chen, Fanqing Meng

**Affiliations:** 1 Department of Pathology, Nanjing Drum Tower Hospital, The Affiliated Hospital of Nanjing University Medical School, Nanjing, China; 2 Department of Respiratory Medicine, Nanjing Drum Tower Hospital, The Affiliated Hospital of Nanjing University Medical School, Nanjing, China

**Keywords:** CCAM, CPAM, Aspergillosis

## Abstract

Congenital cystic adenomatoid malformation (CCAM), also named congenital pulmonary airway malformation (CPAM), is a congenital abnormality of lung which is uncommon in adults. Here we present 2 adult cases of CCAM with unusual clinical and pathologic findings. One case was complicated with aspergillosis which was seldom reported. The other case was suffered bilateral lesions and the patient's mother had been previously radiographically discovered bilateral cystic lesions that CCAM could not be ruled out. A review of currently published related literatures has also been provided.

**Virtual slides:**

The virtual slide(s) for this article can be found here: http://www.diagnosticpathology.diagnomx.eu/vs/6406766736634578.

## Background

Congenital cystic adenomatoid malformation(CCAM) is a developmental anomaly, firstly described as a distinct disease or entity by Ch'in and Tang in 1949 [1]. It was classified into 3 subtypes in 1977 [2], and expanded into 5 types with a new name as congenital pulmonary airway malformation (CPAM) by Stocker in 2002 [3]. 80% to 85% of cases are recognized in the first 2 years of life, adult presentation is uncommon [4]. Most CCAM in adults involve unilateral lobes of the lung, and may be complicated with pulmonary bacterial infections and abscesses [5-7]. Here we described 2 CCAM cases in adults with unusual clinical and pathologic findings, one was complicated with aspergillosis, the other had bilateral lesions with a possible familial association.

## Case presentation

### Case 1

**Figure 1:**
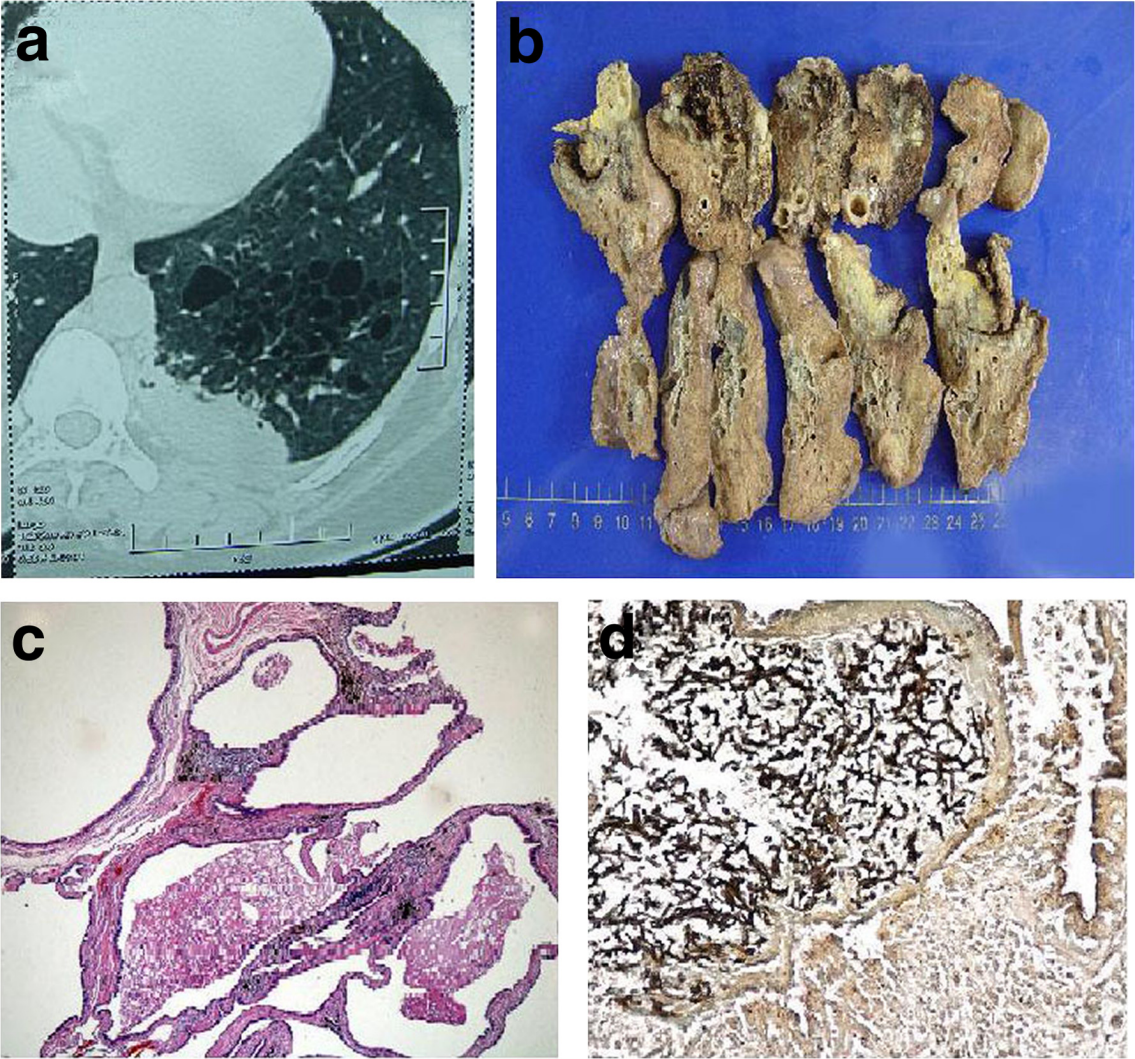
**Patient 1**. **(a) **Computed tomography scan of the chest shows a mass and multiple, cystic changes in the left lower lobe. **(b) **Macroscopic view reveals multi-cystic lesion with partially solid and necrotic area. **(c) **Microscopically, walls of cysts were lined by pseudostratified columnar epithelium and surrounded by fibromuscular bundles. **(d) **Inside the necrotic area, aspergillus hyphae are found and confirmed by GMS stain.

A 26-year-old woman was admitted to hospital because of fever and chest pain with a persistent cough of 1 week's duration. Computed tomography scan of the chest shows a mass and multiple, cystic changes in the left lower lobe (Figure 1, a). As had no benefit from antibiotic therapies, the patient underwent lobectomy. Macroscopic examination revealed a 5 cm*5 cm*2.5 cm mass with plurilocular thin-walled small cysts of uniform size (0.5 cm to 1.5 cm in diameter) (Figure 1, b). Microscopically, the cysts were lined by cuboidal to columnar epithelium and the cystic walls were infiltrated by a large number of inflammatory cells in some area. Exudates and necrotic debris filled in some dilated cysts constituted the solid areas appeared on the CT scan. A significant discovery was that aspergillus hyphae were detected within the lesion and highlighted by Grocott's methenamine silver (GMS) stain (Figure 1, c, d). Both histological and radiological findings were consistent with type II CCAM according to Stocker's classification [2]. The postoperative recovery of the patient was uneventful 4 years after surgery.

### Case 2

**Figure 2:**
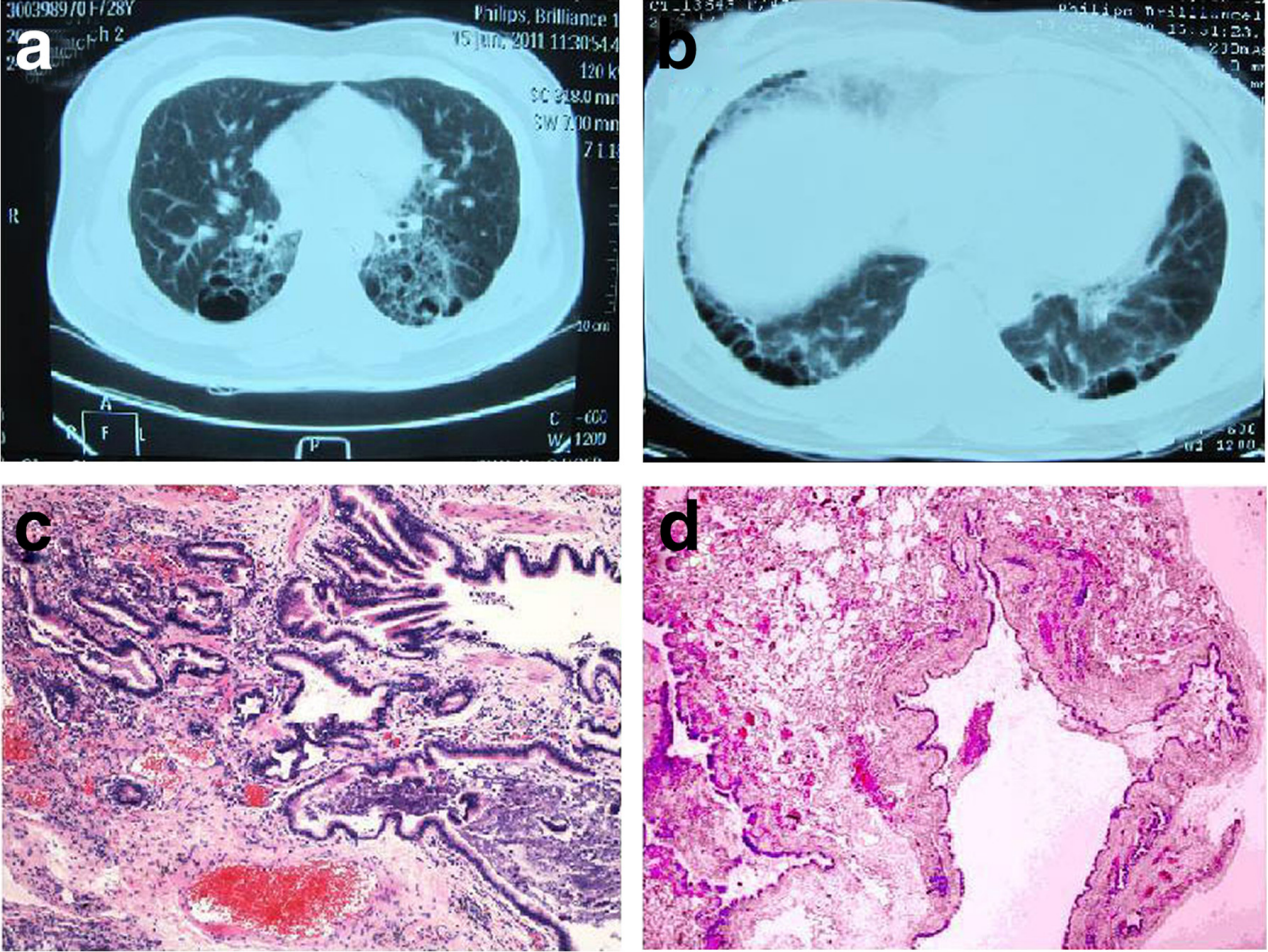
**Patient 2. (a) **Computed tomographic scan of the chest shows multiple, bilateral lung cysts in the lower lobse. **(b) **Computed tomographic scan of the chest shows subpleural predominant cysts in the lower lobse. **(c,d)**The microscopic view revealed adenomatoid proliferated broncholes infiltrated by inflammatory cells.

A 28-year-old woman had a complaint of intermittent nonproductive cough of 10 days' duration, accompanied with mild dyspnea, chest pain and asthma. Her previous CT scan showed multicystic lesions (0.5 cm to 3 cm in diameter) in bilateral lower lobes of the lung beneath the pleura, with peripheral lung tissue showing diffuse grid-like opacity (Figure 2, a). The patient also provided an important history that her mother had been previously discovered multicystic lesions in both lungs on the CT scan that CCAM could not be totally excluded (Figure 2, b). Diagnosed as "interstitial pneumonia with bulla formation" by the clinicians, the patient was hospitalized for further tests. On physical examination, a few crackles were heard over lower part of the chest wall. Pulmonary function examination showed a mild to moderate decrease of mixed type ventilation function and diffusion function. In order to make a definte diagnosis, the clinicians performed an open lung biopsy. Histologically, the lesion was consisted of cysts that were lined by pseudostratified ciliated columnar epithelium (Figure 2, c, d). These findings were in accordance with type I CCAM [8]. Since the patient had a special family history, the possibility of certain genetic association was taken into account. Blood sampling was performed for karyotype analysis, but no remarkable result was found. The patient was treated with symptomatic treatment and showed improvement in her condition. She remained asymptomatic till we contacted her for follow-up at the last time.

## Discussion

The classical histological change of CCAM is that normal pulmonary alveoli are replaced by cysts composed of adenomatoid hyperplastic bronchioles. Based on the size and number of the cysts, this lesion was initially classified into 3 groups [2]. All types are intrinsically the same lesion, but vary on the radiological presentation. Type I CCAM accounts for 70% of cases, is characterized by single or multiple cysts more than 3 cm in diameter lined by pseudostratified ciliated columnar epithelium, along with mucous cells which are considered to potentially mutant to adenocarcinoma [9]. Type II lesion is consisted of multiple terminal bronchiolar-like uniform cysts smaller than 2 cm in diameter, lined by cuboidal to columnar epithelium. Type III CCAM usually involves an entire lobe of lung and has a spongy-like appearance, constructed by bulk gland-like structures. Another 2 types were proposed in 2002 [3]. The new classification system with added type 0 and IV was not widely applied because type 0 is very difficult to differentiate from bronchogenic cyst, and the similarities between type IV and cystic pluropulmonary blastoma may result in misdiagnosis [5]. Late-onset CCAM in adults may be more complicated on radiographic images due to recurrent infections. In this report, case 1 was classified into Stocker's type II CCAM for its uniform size of the cysts and regular proliferation pattern of the bronchiolar epithelium (Figure 1, b, c). Case 2 met the diagnostic criteria of type I for its distinct pathology change that multicysts, containing at least one large cyst larger than 3 cm in diameter with smaller ones in its periphery, were lined by bronchial epithelium with discontinuous smooth muscle within the cystic walls (Figure 2, a, c).

In our report, aspergillus hyphea confirmed by GMS stain were discovered in case 1 (Figure 1, d), which is extremely rare in CCAM cases. By far, bacterial infection is the most frequently reported CCAM complication which causes acute fever and lung abscess [6]. To our knowledge, an aspergilloma within CCAM have been previously documented only in one case [10]. Presentations of aspergillosis may be varied from that of bacterial infection. It may either produce a fulminant invasive pulmonary infection or quietly coexisting for year in the human host without symptoms. Aspergillosis may display cystic cavities or one solid mass on the CT scan that resemble lung tumor or tuberculoma. In this case, aspergillus caused localized tissue damage and produced massive exudates and necrotic debris that constituted areas of consolidation mixed into the cystic lesion, making a complex CT scan image. However, multicysts with uniform size and adenomatoid hyperplastic bronchial structures revealed by gross and histological examination led to the diagnosis of cystic malformation.

In case 2 we presented with, the lesion involving bilateral lobes of the lung is also uncommonly encountered. In CCAM associated literatures, a few bilateral CCAM cases in adult patients have been reported [11-13]. Bilateral CCAM may appear like interstitial pneumonia because of similar CT scan presentations showing grid-like opacity through the entire lung fields. The extensive involvement of the lesion increased risk and difficulty of the surgery. Therefore, most patients with such lesions are treated with conservative treatment after diagnosis confirmed by lung biopsy. In this report, the patient of case 2 was initially diagnosed as "interstitial pneumonia with bulla formation", due to the ventilating dysfunction associated clinical presentations and the thin-walled multicysitc lesion with fibrosis-like change of the peripheral lung tissue. However, an open lung biopsy revealed proliferative malformation rather than interstitial pneumonia. It is noticeable that the patient's mother had also been previously discovered multicystic lesions in bilateral lower lobes of the lung, indicated that the disease might have some kind of familial relevance. Up till now, the exact mechanisms of CCAM remain unknown, but Roberts et al. suggested that this lesion might be familial and related to chromosome abnormalities [14]. Although the result of karyotype analysis in this case was unremarkable, the possibility that this congenital lesion might be heritable or related to chromosome or genetic malfunction could not be completely ruled out.

## Conclusions

CCAM in adults may be accompanied by aspergillus infection and occur in bilateral lobes of lung. The clinical and radiologic findings vary, thus pathological examinations are required to make definite diagnosis on cystic lesion of lung. In addition, the familial history of the patient of case 2 indicated a possibility of familial relevance of this disease. But further study is required to certify this supposition.

## Consent

Written informed consent was obtained from the patients for publication of this Case Report and any accompanying images. A copy of the written consent is available for review by the Editor-in-Chief of this journal.

## Competing interests

The authors declare that they have no competing interests.

## Authors' contributions

Meng FQ Feng AN, and Zhang YF designed the study and wrote the manuscript. Cai HR and Chen LL collected the patients' clinical information. Sun Q obtained the follow-up data and wrote the manuscript. All authors have read and approved the final manuscript.
